# Sono‐Mechanogenetics: Linking Ultrasound Physics With Cellular Mechanobiology

**DOI:** 10.1002/advs.75167

**Published:** 2026-04-13

**Authors:** Yunjia Qu, Fan Wei, Chi Woo Yoon, Qifa Zhou, Yingxiao Wang

**Affiliations:** ^1^ The Alfred E. Mann Department of Biomedical Engineering Viterbi School of Engineering University of Southern California Los Angeles California USA; ^2^ Department of Ophthalmology Roski Eye Institute Keck School of Medicine University of Southern California Los Angeles California USA

## Abstract

Sono‐mechanogenetics aims to achieve remote, noninvasive control of cellular behavior by coupling focused ultrasound with genetically specified biological responses mediated through mechanotransduction pathways. Although recent studies have demonstrated diverse proof‐of‐concept applications, progress in the field has largely emphasized actuator discovery and application‐driven demonstrations, often treating ultrasound as a black‐box stimulus and mechanosensitive elements as isolated sensors. In this review, we seek to reframe sono‐mechanogenetics through the combined lenses of ultrasound physics and cellular mechanobiology. We first describe how ultrasound delivers programmable mechanical energy through distinct deformation modes, and how these physical inputs intersect with biological force‐sensing networks. We then outline core mechanotransduction pathways spanning the extracellular matrix (ECM), membrane, cytoskeleton, and nucleus, and discuss how these systems naturally sense, integrate, and transduce mechanical information. Building on this foundation, we specifically introduce current applications in neural modulation and immunotherapy, emphasizing the underlying mechanical perturbations rather than application‐specific outcomes. Finally, we discuss practical constraints and future directions, highlighting how mechanobiological principles can guide the rational design of next‐generation sono‐mechanogenetic systems. Together, this review aims to provide a focused overview of the field from empirical activation toward mechanistically informed and predictive control.

## Graphical abstract

Sono‐mechanogenetics links ultrasound physics with cellular mechanotransduction to enable noninvasive control of engineered biological systems. Acoustic forces generate distinct deformation modes that activate intracellular signaling pathways, which can be coupled to synthetic gene circuits to regulate diverse cellular functions, including gene expression, immune responses, secretion, and cell state transitions.

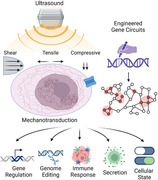

## 1 Introduction

For decades, ultrasound has served as a cornerstone of modern medicine. Its ability to propagate deep within tissue noninvasively has made it indispensable for imaging [1] and an increasingly powerful tool for therapeutic ablation, drug delivery, and neuromodulation [2]. Yet, these conventional approaches face a fundamental limitation: they rely on the intrinsic bioeffects of acoustic energy, such as heating, cavitation, or bulk mechanical stress, which often lack molecular specificity.

Sonogenetics redefines this interaction [3, 4]. By introducing genetic modifications that render specific cells responsive to acoustic stimuli, this approach combines the depth and penetration power of ultrasound with the precision of genetics. The field was formally inaugurated by landmark studies demonstrating that heterologous expression of the TRP‐4 channel in C. elegans [3] could sensitize specific neurons to low‐intensity ultrasound, providing a non‐invasive alternative to optogenetics. This was followed by the adaptation of other robust actuators, such as the large‐conductance mechanosensitive channel (MscL) [5], further establishing that genetic sensitization is a viable strategy for remote cellular control. This convergence allows for the remote regulation of defined molecular pathways deep inside the body, offering a solution to the penetration‐depth limits that constrain optical technologies like optogenetics. Landmark studies have already demonstrated the immense potential of such a strategy, capturing the attention of both biomedical and engineering communities.

In essence, sonogenetics integrates two key elements: the physics of ultrasound, which delivers precisely tunable acoustic energy, and the biological interface, composed of molecular sensors or genetic circuits that translate acoustic cues into defined cellular outputs. Sonogenetic strategies can be broadly categorized by their dominant physical modality. Sono‐thermogenetics utilizes ultrasound‐induced heating to activate temperature‐responsive genetic systems, whereas sono‐mechanogenetics focuses on the mechanical forces produced by ultrasound to engage mechanosensitive pathways. Because ultrasound is, at its core, a form of mechanical energy, this review focuses on sono‐mechanogenetics, examining how ultrasound‐generated forces interact with biological systems to enable specific genetic control. For readers interested in a broader overview of the field or the specific development of sono‐thermogenetic systems, we point toward several excellent recent reviews [4, 6, 7].

Despite these advances, the field of sono‐mechanogenetics remains in an early stage of development. While the feasibility of ultrasound‐mediated cellular control has been convincingly demonstrated, the diversity of available genetic tools and the consistency of stimulation methods remain limited compared with more mature technologies such as optogenetics or chemogenetics. To advance toward reliable and broad applications, it is essential to revisit the fundamentals, both the physics of how ultrasound generates and transmits mechanical energy and the biology of how cells and tissues sense and transduce these forces. Without this mechanistic understanding, further tool development and therapeutic translation may remain empirical and fragmented.

Importantly, most existing discussions of sono‐mechanogenetics have emphasized the identification of ultrasound‐responsive actuators or proof‐of‐concept applications, often treating ultrasound as a black‐box stimulus and mechanosensitive proteins as isolated sensors. In contrast, decades of mechanobiology research have established that mechanical information is sensed, integrated, and stored through distributed, multiscale networks spanning the extracellular matrix (ECM)‐membrane interface, cytoskeleton, nucleus, and chromatin. Bridging this gap requires reframing ultrasound not merely as a trigger, but as a source of programmable mechanical energy that engages endogenous mechanotransduction networks across molecular, cellular, and tissue scales.

This review aims to integrate acoustic physics with cellular mechanobiology by examining how ultrasound‐generated mechanical energy is sensed, transmitted, and decoded by cells. We begin by defining the physical principles governing ultrasound propagation and force generation, and then align these with the core mechanotransduction mechanisms, from the extracellular matrix to the nucleus. By treating these perspectives as parts of a single continuum, we seek to provide a conceptual blueprint for the future of sono‐mechanogenetics. The goal is not only to summarize recent advances, but to move beyond stimulus–response demonstrations toward mechanistically guided control, where acoustic inputs are rationally tuned to engage specific signaling networks and cellular states with genetic precision.

## 2 Ultrasound Physics: A Mechanical Interface With Living Systems

Ultrasound occupies a unique position among physical modalities used to interact with biological systems. As a propagating pressure wave, ultrasound delivers mechanical energy deep within tissues in a noninvasive and highly controllable manner. Unlike optical or chemical stimuli, which are often limited by penetration depth or diffusion, ultrasound enables the remote delivery of mechanical cues across multiple spatial and temporal scales. For sono‐mechanogenetics, this capacity positions ultrasound not merely as an external trigger, but as a physical interface that modifies the mechanical forces experienced by cells in vivo. Understanding how ultrasound generates and distributes mechanical forces is therefore essential for linking its physical properties to cellular responses.

Rather than providing a comprehensive treatment of acoustic theory, this section focuses on the physical aspects of ultrasound most relevant to cellular and tissue‐level mechanotransduction. In particular, we emphasize how ultrasound generates distinct deformation modes, including pressure oscillations, displacement, shear, and bubble‐mediated stress, that interact with biological structures in context‐dependent ways. Framing ultrasound in terms of these mechanical effects provides a basis for connecting acoustic parameters with the mechanotransduction pathways discussed in subsequent sections, and for interpreting heterogeneous biological responses across experimental systems.

### 2.1 Ultrasound as a Mechanical Wave

Ultrasound is a longitudinal mechanical wave that propagates through a medium via alternating phases of compression and rarefaction [8]. These oscillatory pressure fluctuations induce particle displacement and cyclic deformation of the surrounding material. In biological tissues, such deformations occur across a wide range of length scales, from nanometer‐scale membrane undulations to micrometer‐scale cellular and tissue displacements [9]. The frequency of ultrasound determines its wavelength and thus the spatial scale of mechanical interaction. Higher frequencies provide finer spatial resolution but experience greater attenuation, whereas lower frequencies penetrate deeper while distributing mechanical energy over larger volumes. These trade‐offs are not merely technical constraints; they directly influence which biological structures experience deformation and how deformation gradients form within tissue.

Unlike static or quasi‐static mechanical perturbations commonly used in mechanobiology, ultrasound introduces rapid, oscillatory mechanical loading rather than slowly varying or global deformation. At ultrasonic frequencies, tissues experience repeated cycles of compression and rarefaction on microsecond timescales. Even when time‐averaged forces are small, cyclic deformation can modulate membrane tension, alter cytoskeletal prestress, and bias mechanosensitive signaling pathways [10, 11, 12]. This dynamic loading regime distinguishes ultrasound from contact‐based force application and underlies its ability to engage mechanotransduction pathways without direct physical contact.

While individual oscillatory cycles occur on microsecond timescales, they are typically much faster than the characteristic response times of most cellular and molecular processes. As a result, cells are unlikely to directly resolve or track each cycle of the acoustic waveform. Consistent with this, experimental observations often indicate that cellular responses are more strongly influenced by pressure amplitude, pulse structure, and exposure duration than by the carrier frequency itself [13, 14, 15]. This suggests that biological effects are primarily mediated by time‐averaged or cumulative mechanical perturbations, rather than direct tracking of high‐frequency oscillations, in line with broader principles of mechanobiological signal integration [16, 17].

At the same time, alternative mechanistic models have proposed that the lipid bilayer may couple to ultrasonic oscillations at much smaller spatial scales. In particular, intramembrane cavitation or “bilayer sonophore” models suggest that nanometer‐scale membrane oscillations can produce capacitance changes and associated ionic currents, providing a potential route for converting high‐frequency mechanical inputs into electrical signals [18, 19, 20]. These mechanisms have been invoked to explain aspects of ultrasonic neuromodulation, although their relative contribution under typical experimental conditions remains under active investigation.

Together, these perspectives suggest that ultrasound–cell interactions may span multiple regimes, ranging from effective, time‐averaged mechanical perturbations at the cellular scale to localized, high‐frequency membrane dynamics at subcellular scales.

### 2.2 Propagation Media, Coupling, and Boundary Conditions

Because ultrasound is a mechanical wave, its propagation depends critically on the mechanical properties of the surrounding medium, including density and speed of sound [8]. Acoustic energy is strongly reflected at interfaces with large acoustic impedance mismatches, such as air–tissue boundaries. As a result, coupling media such as water or gel are essential for efficient ultrasound transmission in both in vitro and in vivo experiments.

Boundary conditions play a major role in shaping the local acoustic field. Reflections, interference between incident and reflected waves, and the formation of standing waves can substantially redistribute mechanical stress [21]. In confined or layered environments, these effects can locally amplify or suppress deformation in ways that are not captured by nominal exposure parameters.

These considerations underscore a central principle for sono‐mechanogenetics: ultrasound‐induced mechanical cues are not determined solely by transducer output, but by the interaction between the acoustic field and the mechanical architecture of the system. Tissue geometry, material heterogeneity, and experimental configuration collectively define the local stress, strain, and deformation fields experienced by cells, helping explain why nominally similar acoustic settings can yield divergent biological outcomes across model systems.

### 2.3 Transducers, Focusing, and Spatial Control of Mechanical Energy

Ultrasound is typically generated using piezoelectric transducers that convert electrical excitation into mechanical vibration. Transducer design determines operating frequency, bandwidth, and beam geometry, while drive conditions largely control acoustic pressure amplitude [1].

Focused ultrasound concentrates mechanical energy within a confined focal volume, enabling localized delivery of stress and deformation at depth. Single‐element transducers provide fixed focusing determined by geometry or acoustic lenses, whereas array‐based systems enable electronic beam steering and dynamic focusing through phase control of individual elements. These capabilities allow mechanical energy to be spatially patterned in three dimensions without physical movement of the source.

From a mechanobiological perspective, spatial control is important not only for targeting specific tissue regions, but also for shaping gradients of stress and strain. Such gradients are known to influence cytoskeletal organization, nuclear deformation, and collective cell behavior. Ultrasound, therefore provides access to spatially patterned mechanical inputs that are difficult to reproduce using conventional contact‐based loading approaches, particularly in vivo.

### 2.4 Temporal Structure, Exposure Parameters, and Viscoelastic Filtering

Beyond spatial localization, ultrasound offers exceptional control over the temporal structure of mechanical stimulation. Pulse duration determines the length of each mechanical perturbation, pulse repetition frequency (PRF) defines the spacing between perturbations, and duty cycle (DC) specifies the fraction of time ultrasound is actively delivered. Mechanobiology has shown that cells respond not only to force magnitude, but also to the duration, repetition, and history of mechanical inputs [16, 17]. A defining feature of sono‐mechanogenetics is the “temporal mismatch” between microsecond acoustic oscillations (MHz) and the second‐to‐hour timescales of biological signaling. This gap is bridged through the nonlinear extraction of the stimulus envelope via the acoustic radiation force (ARF), which is proportional to the time‐averaged intensity of the wave rather than its instantaneous pressure [22]. Consequently, the cell does not “track” individual pressure cycles; instead, it experiences a quasi‐static mechanical push that follows the lower‐frequency dynamics of the waveform envelope, effectively converting MHz energy into a Hz‐to‐kHz mechanical load [23].

The translation of this force into actual cellular deformation is further shaped by the viscoelastic properties of the tissue or ECM. Unlike purely elastic materials where strain mirrors stress instantaneously, biological tissues exhibit time‐dependent creep and stress relaxation [24, 25]. When an ARF “push” is applied, displacement builds over a characteristic creep phase and recovers only after the force is removed. This mechanical response acts as a low‐pass filter, “stretching” brief acoustic pulses into longer mechanical events. In viscous‐dominated environments, this filtering smooths high‐frequency transients into the lower‐frequency ranges (∼100 Hz) most effective for engaging focal adhesions and gating mechanosensitive ion channels [25].

This temporal hierarchy explains why commonly reported metrics like peak pressure provide only indirect information about biological outcomes. Establishing mechanistically meaningful mappings requires matching the ultrasound pulse train to the characteristic relaxation times of the targeted biological circuit. For instance, varying the PRF while holding the carrier and duty cycle constant can recruit non‐overlapping subsets of neurons [26] or significantly alter calcium response rates [27]. Furthermore, repeated loading can strengthen cytoskeletal tension and induce “mechanical memory” that persists after unloading [17]. In this context, ultrasound is not merely a trigger, but a source of programmable mechanical energy that can be delivered as brief impulses, sustained loading, or complex pulse trains to engage specific mechanotransduction networks with temporal precision.

### 2.5 Ultrasound‐Induced Mechanical Bioeffects

**FIGURE 1:**
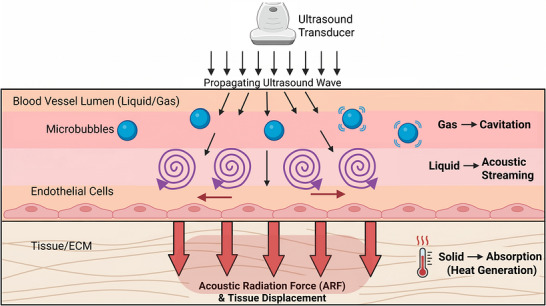
Classification of ultrasound bioeffects by material phase. The schematic depicts a propagating ultrasound wave penetrating a vascularized tissue model. **Gas phase**: Interaction with microbubbles triggers cavitation. **Liquid phase**: Acoustic energy transfer drives bulk fluid motion (acoustic streaming), creating vortex patterns and shear stress at the endothelial boundary. **Solid phase**: The tissue matrix experiences Acoustic Radiation Force (ARF) leading to displacement, alongside thermal effects caused by energy absorption. These distinct mechanical and thermal stimuli are primary drivers of sono‐mechanotransduction. **Disclosure Statement**: Initial conceptual layout and draft visual design were generated with assistance from Google Gemini (accessed January 2026). The figure was subsequently redrawn, refined, arranged, and finalized by the authors using BioRender. All scientific content and visual elements were reviewed and verified by the authors for accuracy and appropriateness.

As ultrasound propagates through tissue, acoustic energy is attenuated primarily through absorption, with scattering contributing to a lesser extent in most soft tissues [8]. Attenuation gives rise to a range of bioeffects, including both mechanical and thermal responses [28]. Because this review focuses on sono‐mechanogenetics, we restrict discussion here to nonthermal mechanical bioeffects that directly deform cells and tissues, and do not cover ultrasound‐induced heating or thermally mediated mechanisms, which have been extensively reviewed elsewhere. From a mechanobiological perspective, these mechanical bioeffects can be viewed as distinct deformation modes, each generating characteristic stress, strain, or shear fields that engage different cellular sensing interfaces (Figure 1). Framing ultrasound in terms of these modes provides a basis for linking acoustic parameters to specific mechanotransduction pathways.

#### 2.5.1 Acoustic Radiation Force

Acoustic radiation force arises from momentum transfer when there is a spatial gradient in acoustic intensity [28, 29]. This force produces steady or slowly varying tissue displacement and is widely regarded as a dominant nonthermal mechanical effect in low‐intensity ultrasound stimulation [29]. Radiation force can deform cells and extracellular matrices over micrometer scales, generating stresses comparable to those used in classical mechanobiology experiments. Because radiation force effects accumulate over time, they are particularly relevant for sustained or repetitive stimulation paradigms, including neuromodulation and mechanogenetic activation, where they can engage integrin‐based adhesions and cytoskeletal tension through micrometer‐scale deformation.

#### 2.5.2 Cavitation and Mechanical Amplification

At sufficiently high peak negative pressures, ultrasound can induce cavitation, characterized by the oscillation or collapse of gas‐filled bubbles [30]. Stable cavitation involves sustained oscillations that can amplify local mechanical stresses, whereas transient cavitation produces rapid bubble collapse and highly energetic events. Cavitation thresholds depend on acoustic parameters and medium properties, motivating the introduction of the Mechanical Index as a rough indicator of cavitation likelihood [9].

From a mechanobiological perspective, cavitation introduces highly localized and nonlinear mechanical stresses, including extreme shear, membrane poration, and ECM disruption [20, 31, 32]. While these effects offer powerful mechanical amplification, they also reduce predictability, presenting both opportunities and challenges for controlled biological modulation, particularly in systems where membrane integrity and local microenvironment are critical.

To improve the coupling between ultrasound and cells and enable precise mechanical amplification, genetically encoded acoustic actuators such as gas vesicles (GVs) have been developed. These protein‐shelled, gas‐filled nanostructures leverage their high compressibility to lower the threshold for ultrasonic actuation. For instance, GVs can significantly enhance low‐intensity ultrasound neuromodulation [33] or be converted by low‐frequency ultrasound into cavitating bubbles to produce localized mechanical effects [34]. Most importantly for transcriptional control, GVs have been shown to amplify ultrasound‐induced Ca^2+^ influx and couple this response to synthetic calcium‐dependent signaling pathways [35]. By serving as programmable mechanical sensitizers, GVs allow for the focused delivery of acoustic radiation forces and stable cavitation directly at the cellular interface, transitioning the field from stochastic cavitation toward genetically targeted mechanical control.

#### 2.5.3 Acoustic Streaming

Acoustic streaming refers to steady fluid motion induced by acoustic energy absorption or by oscillating structures such as bubbles. Streaming generates shear stress at interfaces and can enhance transport in the extracellular environment [36, 37]. Shear stress is a well‐established regulator of cellular behavior, particularly in endothelial [38], epithelial [39], and immune systems [40]. Ultrasound‐induced streaming, therefore, represents an additional, often underappreciated, mode of shear‐based mechanical input that can engage mechanotransduction pathways sensitive to fluid flow and interfacial stress that may contribute to observed biological responses, especially in fluid‐filled or vascularized environments.

Together, these nonthermal bioeffects define the primary mechanical routes by which ultrasound can generate distinct stress, strain, and shear environments at the cellular and tissue scales. These deformation modes provide the physical basis through which ultrasound can engage integrin‐based adhesions, cytoskeletal tension, membrane mechanics, and collective tissue deformation, enabling sono‐mechanogenetic actuation without reliance on temperature elevation.

### 2.6 Defining the Mechanical Regime: Boundary Conditions and Minimal Criteria

To transition sono‐mechanogenetics from an empirical to a predictive science, the field requires a standardized definition of the “primarily mechanical” regime. Because ultrasound is a multi‐modal energy source, mechanical effects must be explicitly decoupled from thermal and cavitational bioeffects. No single parameter set is universally “mechanical” across all biological systems, as heating and cavitation depend heavily on tissue attenuation, geometry, and dissolved gas content. However, establishing concrete boundary conditions remains essential for mechanistic rigor. Tissue heating is primarily governed by the spatial‐peak temporal‐average intensity (I_SPTA_), together with attenuation and exposure duration. In the context of sonogenetics, “primarily mechanical” stimulation typically operates at I_SPTA_ values below 1–2 W/cm^2^, often utilizing low DC (<10%) to allow for thermal relaxation between pulses. A rigorous minimal criterion for mechanical interpretation is a localized temperature rise of ΔT<0.5°C, ensuring the response is not confounded by endogenous heat‐shock proteins or thermosensitive ion channels.

While stable cavitation can amplify mechanical signals, inertial cavitation is generally excluded from the intended mechanical regime due to its potential for non‐specific damage. The Mechanical Index serves as a first‐order indicator of cavitation likelihood; for a study to be classified as primarily mechanical, protocols should ideally maintain an MI below the FDA diagnostic safety limit of 1.9, particularly in the absence of exogenous contrast agents. Consequently, we propose that to establish a “mechanical safe harbor,” studies should meet three reporting standards: (i) explicit quantification of peak negative pressure and I_SPTA_, (ii) evidence of thermal safety through direct measurement or bioheat modeling, and (iii) the use of pulsed waveforms designed to stay below the inertial cavitation threshold. Table 1 contrasts these representative parameter regimes, highlighting the distinct boundaries that separate the mechanical “safe harbor” from thermal‐dominant (hyperthermia) and cavitation‐dominant (histotripsy) applications.

**TABLE 1:** Comparison of representative ultrasound parameter regimes associated with primarily mechanical, heating‐dominant, and cavitation‐dominant bioeffects. The two “primarily mechanical” studies explicitly ruled out substantial heating and cavitation, whereas the hyperthermia and histotripsy examples serve as contrasting thermal and cavitation regimes, respectively. I_SPTA and MI are shown as reported in the original papers or estimated from the published acoustic parameters where not explicitly provided.

Regime	Paper	I_SPTA	MI	Validation
Primarily mechanical	Yoo et al., 2022 [14]	0.375 W/cm^2^ (Estimated)	0.9 (reported)	Heating: fiber‐optic thermometer, negligible (<0.02°C). Cavitation: no bubbles imaged (5Mfps)
	Matsushita et al., 2024 [127]	0.19 W/cm^2^ (reported)	0.11(Estimated)	Heating: thermocouple, minimal (<0.8°C). Cavitation: not detected by negative starch–iodine assay
Heating/hyperthermia	Partanen et al., 2012 [204]	400 W/cm^2^ (reported)	3.07 (Estimated)	Cavitation: not detected
Cavitation	Lin et al., 2014 [205]	∼0.08–0.87 W/cm^2^ (Estimated)	34.6–114.1 (Estimated)	Cavitation: dense bubble clouds and lesions were generated

### 2.7 In Vivo and In Vitro Context Dependence

Ultrasound‐induced mechanical effects differ substantially between in vivo and in vitro environments. In vivo, tissue heterogeneity, interfaces, motion (e.g., respiration), and structures such as bone can distort acoustic fields and redistribute mechanical stress. Imaging‐guided approaches, including MRI‐based thermometry [41, 42] and ultrasound elastography [43, 44, 45], are often employed to monitor and control these effects. In vitro systems offer greater experimental control but introduce artificial boundary conditions that can exaggerate standing waves or alter force distribution [11, 28]. While such models are invaluable for isolating specific mechanical phenomena, they can also generate mechanical inputs that differ significantly from those in physiological settings.

As a result, identical acoustic parameters can produce different stress, strain, and deformation fields across experimental systems. Careful consideration of mechanical context is therefore essential for interpreting ultrasound‐induced biological responses and for translating findings between in vitro and in vivo environments.

### 2.8 Ultrasound Physics as a Foundation for Sono‐Mechanogenetics

Together, these physical principles establish ultrasound as a versatile source of programmable mechanical energy. Ultrasound can deliver spatiotemporally structured forces, generate multiple deformation modes, and interact with complex tissues in a noninvasive manner. These features define a multidimensional mechanical input space, shaped by spatial focusing, temporal patterning, deformation mode, and physical context. However, acoustic parameters alone do not uniquely determine biological outcome. The mechanical inputs experienced by cells depend on how acoustic fields interact with tissue architecture and boundary conditions, while mechanobiology governs how these inputs are sensed, integrated, and remembered. Bridging these domains is therefore essential for advancing sono‐mechanogenetics from empirical demonstration toward mechanistically guided design.

Building on these physical foundations, the next question is how ultrasound‐generated mechanical inputs are sensed and interpreted by cells. In the following section, we draw on insights from mechanobiology to examine how cells detect, integrate, and respond to mechanical inputs, establishing a foundation for interpreting ultrasound‐driven biological responses.

## 3 Key Mechanotransduction Mechanisms and Components

**FIGURE 2:**
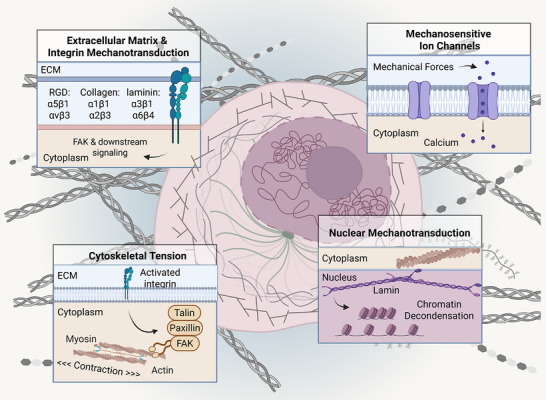
Core mechanotransduction interfaces relevant to sono‐mechanogenetics. Schematic overview of major cellular pathways that convert mechanical cues into biochemical signaling and gene regulation. Mechanical information can be sensed at the cell–ECM interface through integrins and focal adhesions (top left), where ligand engagement (e.g., RGD‐, collagen‐, and laminin‐binding integrins) triggers FAK‐centered signaling. Forces are transmitted through the actomyosin cytoskeleton to generate cytoskeletal tension and reinforce adhesions via force‐sensitive adaptor proteins (e.g., talin, paxillin) (bottom left). In parallel, membrane deformation can gate mechanosensitive ion channels, producing rapid Ca^2^^+^ influx and downstream signaling (top right). These mechanical inputs also propagate to the nucleus through cytoskeletal–nuclear coupling, engaging nuclear mechanotransduction via the lamina and promoting changes in chromatin organization (e.g., decondensation) that shape transcriptional programs (bottom right). Together, these interconnected modules provide a mechanistic “map” of biological entry points through which ultrasound‐generated forces may be sensed, integrated, and converted into functional cellular outputs.

Cells constantly sense and respond to a variety of physical cues within their microenvironment, ranging from ECM stiffness and viscoelasticity to shear stress, compression, and cyclic stretch through specialized mechanotransduction machinery that converts physical forces into biochemical signals. Mechanical information is not processed by a single sensor but distributed across integrin‐based adhesions, cytoskeletal networks, the nucleus, and mechanosensitive ion channels, enabling force transmission across pico‐ to nano‐newton scales. At the cell‐matrix interface, integrins and focal adhesions initiate force transmission. These signals are propagated through the cytoskeleton to the nucleus, where the nuclear lamina and chromatin respond to mechanical deformation. At higher levels of organization, tissue‐scale forces emerge from cell–cell junctions, collective migration, and ECM architecture, providing long‐range mechanical cues that shape morphogenesis and disease. Together, these pathways enable cells to decode not only force magnitude, but also temporal patterning and mechanical history (Figure 2).

Ultrasound delivers oscillatory mechanical energy within force and deformation regimes relevant to these pathways, thus mechanobiology provides a necessary framework for understanding sono‐mechanogenetic control. Acoustic pressure waves, radiation forces, cavitation‐induced strain, and fluid motion can generate membrane tension, matrix deformation, and cellular displacement across a wide range of spatial and temporal scales. In this section, we focus on the core mechanotransduction mechanisms most relevant to ultrasound‐induced mechanical stimulation and discuss how these biological interfaces can serve as entry points for sono‐mechanogenetic actuation.

### 3.1 Extracellular Matrix and Integrin Mechanotransduction

Cells reside within the ECM, with mechanical cues directing behaviors such as differentiation, migration, and proliferation [46, 47]. Within this context, cells exert pushing and pulling forces on the ECM, while the ECM resists deformation according to its stiffness [48, 49]. Physiological ECM stiffness ranges from hundreds to several kilos of Pascals (Pa) in soft tissues like brain, adipose, and breast tissues, to tens kPa in muscles and bones [48, 49]. Beyond elasticity, the ECM exhibits viscoelastic behavior characterized by stress relaxation and creep under sustained loading [16, 46]. Because ultrasound imposes cyclic rather than static deformation, ECM viscoelasticity critically shapes how acoustic forces are transmitted and dissipated at the cell interface.

Mechanical coupling between cells and the ECM is mediated by integrins, heterodimeric αβ transmembrane receptors that link extracellular ligands to the actin cytoskeleton via focal adhesions [46, 47, 50]. Integrins act as force‐bearing molecular clutches with ligand‐ and force‐dependent behaviors, including fibronectin‐binding integrins α5β1 and αvβ3 that form ligand bonds [51, 52, 53, 54, 55] and Collagen‐binding integrins such as α2β1 sustain higher rupture [56, 57, 58].

Integrin engagement drives the assembly of focal adhesions (FAs), multiprotein complexes that mechanically couple the ECM to actin stress fibers [59]. Nascent adhesions mature into elongated structures (∼3–10 µm) capable of integrating forces from individual integrin bonds (∼20–160 pN) into cellular‐scale traction forces on the order of tens of nanonewtons [60, 61]. Talin unfolds under tension to expose vinculin‐binding sites, reinforcing integrin–actin coupling [60]. Vinculin stabilizes force transmission through direct actin binding [62], while other adaptors such as filamin and α‐actinin exhibit force‐dependent unfolding or rupture [63]. Paxillin integrates mechanical inputs with signaling pathways that regulate adhesion turnover and migration [64]. Focal adhesion kinase (FAK) also serves as a central signaling hub within FAs. Upon integrin clustering and force transmission, FAK undergoes autophosphorylation at Tyr397, which recruits Src‐family kinases to initiate downstream PI3K‐Akt, MAPK/ERK, and Rho GTPase pathways, thereby coordinating cytoskeletal contractility, adhesion turnover, and survival signaling [65, 66, 67].

From a sono‐mechanogenetic perspective, integrin‐based adhesions are particularly sensitive to cyclic matrix deformation generated by acoustic radiation force, cavitation‐induced strain, or acoustic streaming. These forces typically occur on millisecond–second pulse timescales and may repeatedly load integrin–ligand bonds near their rupture thresholds, suggesting that pulsed ultrasound can bias adhesion signaling without structural disruption.

### 3.2 Cytoskeletal Tension and Nuclear Mechanotransduction

Once mechanical signals are sensed at integrin‐based adhesions, the cytoskeleton provides the principal force propagation and conversion into biochemical responses through connections to the nucleus. The actin cytoskeleton, organized into stress fibers, serves as a scaffold that transmits extracellular forces while generating intracellular contractility through non‐muscle myosin II (NMII) activity [68]. Stress fibers anchored at focal adhesions channel tension across the cell [69, 70]. Integrin engagement activates RhoA–ROCK–MLC2 signaling, increasing myosin light chain phosphorylation, NMII activity, and stress fiber tension, which stabilizes focal adhesions through FAK–Src–paxillin signaling [71]. Under sustained loading, actin‐associated proteins such as zyxin and VASP reinforce strained filaments, while repeated force induces persistent cytoskeletal remodeling that can persist after unloading (“mechanical memory”) [72, 73, 74, 75]. Other filament systems act cooperatively: intermediate filaments such as vimentin and keratins dissipate mechanical stress and provide viscoelastic resistance [76], while microtubule acetylation stabilizes focal adhesions and promotes contractility [77].

Cytoskeletal forces converge on the nucleus through the linker of nucleoskeleton and cytoskeleton (LINC) complex, which transmits tension across the nuclear envelope [78, 79]. KASH (Klarsicht, ANC‐1, and Syne Homology)‐domain proteins (nesprins) connect cytoskeletal filaments to SUN (Sad1 and UNC‐84)‐domain proteins in the inner nuclear membrane, forming a continuous mechanical linkage to the nuclear lamina, composed primarily of lamins A and C. Lamin A/C levels scale with matrix stiffness, increasing on rigid substrates and enhancing nuclear stiffness and resistance to deformation, while softer environments promote nuclear fluidity and chromatin relaxation [48]. Lamins A/C interact with emerin, lamin B receptor (LBR), and barrier‐to‐autointegration factor (BAF) to tether lamina‐associated domains (LADs) and maintain heterochromatin organization [80]. Force transmission through the LINC–lamin axis disrupts LAD tethering, increases chromatin accessibility of mechanoresponsive genes, and is reversed upon relaxation, consistent with Polycomb‐mediated mechanoregulation [81, 82, 83].

Mechanical deformation of the nucleus propagates into chromatin, where changes in nucleosome spacing, histone modifications, and transcription factor accessibility translate physical inputs into transcriptional outputs. Direct mechanical deformation promotes chromatin decondensation and histone acetylation (e.g., H3K9ac) while reducing repressive marks (e.g., H3K27me3) [84, 85]. Mechanosensitive transcriptional regulators including YAP/TAZ and MRTF‐SRF accumulate in the nucleus in response to sustained tension, coupling cytoskeletal contractility to gene expression programs that govern proliferation, differentiation, and migration [86, 87]. These nuclear and chromatin states can persist after force withdrawal, establishing an epigenetic form of mechanomemory encoded by lamin stabilization and chromatin accessibility [88]. In ultrasound stimulation, cytoskeletal and nuclear mechanotransduction are primarily engaged by cell‐scale deformation generated by acoustic radiation force or acoustic streaming. Because these pathways integrate forces over seconds to minutes, effective sono‐mechanogenetic actuation may require repeated or temporally patterned stimulation rather than single acoustic pulses.

### 3.3 Mechanosensitive Ion Channels

When cells experience membrane deformation, mechanosensitive ion channels are among the first layers to sense and initiate downstream pathways. Such mechanical deformations can be caused by tension, ECM stiffness, stretching, osmotic shock, externally applied mechanical perturbations, including ultrasound.

Mechanosensitive channels gate through force‐from‐lipid or force‐from‐filament mechanisms, directly coupling membrane mechanics to ion flux and intracellular signaling [89, 90, 91, 92]. Their fast kinetics and reversibility make them well‐suited for dynamic mechanical stimulation.

Piezo1 is a broadly expressed nonselective cation channel activated by stretch, stiffness, osmotic pressure, or ultrasound, mediating Ca^2^^+^ influx that regulates inflammation, migration, proliferation, and differentiation [93, 94, 95, 96]. Piezo‐1 mediated Ca^2^^+^ entry directly activates transcriptional regulators, such as NFAT, YAP, and β‐catenin, linking mechanical inputs to gene expression programs [97, 98]. Its homolog Piezo2 serves as the principal mechanotransducer in sensory neurons for light touch, vibration, and proprioception in sensory neurons [99]. Together, Piezo channels act as central molecular conduits for ultrasound‐induced membrane stress.

The transient receptor potential (TRP) channel family represents a second major class of mechanosensitive channels, including TRPA1, TRPV4, and TRPM7. Unlike Piezo channels, many TRPs rely on hybrid gating mechanisms that couple bilayer tension with cytoskeletal interactions. TRPA1 is activated by membrane stress, enabling sensory neurons to integrate mechanical and chemical stimuli to drive Ca^2^^+^ influx underlying nociception and inflammatory pain [100, 101]. TRPV4 transduces osmotic and shear stress into cytoskeletal remodeling and transcriptional signaling through RhoA/ROCK, MRTF‐A, YAP/TAZ, and AKT pathways, regulating vascular and epithelial mechanics [102, 103, 104]. TRPM7 is a stretch and swelling‐activated channel‐kinase, where it conducts Ca^2^^+^ and Mg^2^^+^ while directly regulating cytoskeletal dynamics through phosphorylation of substrates such as myosin IIA heavy chain [105]. K2P potassium channels, including TREK‐1 (KCNK2) and TRAAK (KCNK4), are directly gated by bilayer tension and respond to stretch, pressure, temperature, and lipid composition [106, 107, 108, 109]. Interestingly, TREK‐1 acts as a mechano‐gated K^+^ channel that dampens mechanical sensitivity, since mice lacking TREK‐1 show stronger responses to light touch and mechanical pain [110]. Beyond mammalian systems, the bacterial mechanosensitive channel MscL exemplifies force‐from‐lipid gating, opening at high membrane tension to prevent lysis during osmotic downshock and serving as an engineered actuator for ultrasound‐triggered ionic influx and gene regulation in mammalian cells [89, 111, 112].

A growing body of work directly demonstrates the ultrasound sensitivity of multiple mechanosensitive channels. K2P channels exhibit ultrasound‐modulated currents driven by membrane tension, with responses that are rapid, reversible, and pharmacologically controllable [113, 114]. Piezo1 has emerged as a central mediator of ultrasound‐induced signaling across cell types, including neurons, fibroblasts, and osteoblasts, and has been successfully integrated into engineered gene circuits to enable ultrasound‐responsive CAR expression in T cells for precise immunomodulation [98, 115, 116, 117]. TRP family members further extend this paradigm: TRPA1 functions as a low‐intensity ultrasound sensor in astrocytes and neurons, triggering Ca^2^^+^ influx and downstream signaling, while TRPC1 and related channels contribute to ultrasound‐evoked calcium dynamics across multiple tissues [14, 118, 119, 120]. Engineered systems further highlight the tunability of channel‐based actuation. Mutant variants of the bacterial channel MscL (e.g., G22S, I92L) enable low‐pressure, millisecond‐scale activation and have been applied for in vivo neuromodulation, including restoration of visual function under focused ultrasound [121, 122, 123]. MEC‐4 was implicated through ultrasound‐evoked behavioral and neuronal responses in C. elegans, with loss‐of‐function mutants showing reduced sensitivity [124]. TREK‐2 has been shown to respond in a steep, switch‐like manner within a narrow range of ultrasound perturbations, highlighting its potential as a tunable actuator [125]. In mammalian systems, ASIC1a and TRPC6 have also been implicated: ASIC1a activation induces rapid ERK phosphorylation in the mouse brain following ultrasound stimulation [126], while a pharmacological blocker of TRPC6 via intracerebroventricular delivery significantly reduces ultrasound‐evoked neuronal firing in the cerebral [127].

Collectively, mechanosensitive ion channels are particularly responsive to rapid membrane tension oscillations generated directly by the ultrasound pressure field. These oscillations occur on microsecond–millisecond timescales corresponding to acoustic cycles, explaining why channel‐based sono‐mechanogenetic systems often employ short pulses and moderate duty cycles to maximize membrane deformation while minimizing heating or bulk tissue displacement.

### 3.4 Mechanotransduction at the Cell‐Cell Interface and Tissue Structure

Beyond the single‐cell scale, mechanical cues are integrated and propagated across tissues through cell–cell junctions, collective cell behavior, and extracellular matrix organization. At this level, tissue mechanics emerge from coordinated cellular activity rather than isolated cytoskeletal contractions. Intercellular junctions and ECM networks form a mechanically continuous scaffold that distributes tension and enables tissues to sense, adapt, and remodel in response to internal or external stress. Cell–cell junctions transmit and balance forces between neighboring cells, integrating individual cytoskeletal tension into cohesive tissue mechanics. Adherens junctions, desmosomes, and tight junctions couple epithelial and endothelial cells into mechanically continuous assemblies. In particular, cadherin–catenin complexes link cell–cell adhesion to the actin cytoskeleton and respond to mechanical load: α‐catenin undergoes force‐induced conformational changes that expose vinculin‐binding sites, reinforcing adhesion and stabilizing intercellular force transmission [128, 129]. Desmosomes distribute tensile stress through intermediate filament networks, while tight junctions interface with actomyosin contractility to regulate epithelial tension homeostasis [39, 130].

As forces propagate through junctional networks, mechanical coordination extends beyond immediate neighbors to entire cell collectives. During collective migration, leader cells generate traction forces that are transmitted rearward via adherens junctions and the actin cytoskeleton to follower cells [131]. Such coordinated mechanics govern wound repair, morphogenesis, and branching processes, while interstitial fluid pressure and growth‐induced solid stress compress tissues, altering perfusion, transport, and signaling [132, 133, 134, 135, 136]. Through these multiscale interactions, tissues integrate local cellular forces into emergent mechanical states that regulate homeostasis, regeneration, and disease progression [137]. At the tissue scale, ultrasound can generate distributed deformation through radiation force, pressure gradients, and fluid motion acting across multicellular assemblies on millisecond–second timescales. These regimes suggest that sono‐mechanogenetic stimulation may preferentially influence collective tissue behaviors such as barrier permeability, morphogenesis, or coordinated cell migration rather than isolated single‐cell responses. Together, these mechanotransduction pathways provide a vocabulary for describing how mechanical information is processed by living systems, a prerequisite for transforming sono‐mechanogenetics from empirical stimulation into a rationally designed modality. To facilitate the rational design of sono‐mechanogenetic systems, Table 2 summarizes the characteristic force thresholds and deformation regimes of these components, providing a quantitative roadmap for matching acoustic inputs to specific biological sensors.

**TABLE 2:** Core mechanotransduction components, force regimes, and relevance to sono‐mechanogenetic actuation.

Mechanotransduction level	Key components	Typical force/deformation regime	Mechanistic role	Citation
ECM–integrin interface	Integrins (α5β1, αvβ3, α2β1)	∼40–160 pN per bond	Force‐bearing molecular clutches that sense matrix stiffness and cyclic deformation	[51, 52, 53, 54, 55, 56, 57, 58]
Focal adhesions	Talin, vinculin, paxillin, FAK	∼30–50 pN (talin unfolding)	Integrates molecular‐scale forces into biochemical signaling and cytoskeletal reinforcement	[60]
Actin cytoskeleton	Stress fibers, non‐muscle myosin II	Tens of nN per cell	Propagates and amplifies mechanical inputs across the cell	[60, 61]
Intermediate filaments	Vimentin, keratins	Viscoelastic damping over seconds–minutes	Dissipates stress and buffers deformation	[76]
Microtubules	Acetylated microtubules	Load‐dependent stabilization	Coordinates adhesion maturation and contractility	[77]
Nucleus–cytoskeleton coupling	LINC complex (nesprins, SUN proteins)	Nuclear strain under cytoskeletal tension	Transmits force to the nucleus	[78, 79]
Nuclear lamina	Lamin A/C	Stiffness‐dependent scaling	Controls nuclear stiffness and deformation	[48]
Chromatin	LADs, histone modifications	Nucleosome‐scale deformation	Converts mechanical strain into transcriptional accessibility	[79, 84, 85]
Mechanosensitive ion channels	Piezo1, Piezo2	Rapid gating under membrane tension	Fast Ca^2^^+^ influx and signaling initiation	[93, 94, 95, 96, 97, 98, 99]
	TRPA1, TRPV4, TRPM7	Hybrid lipid–cytoskeleton gating	Integrates mechanical and biochemical cues	[100, 101, 102, 103, 104, 105]
	TREK‐1, TRAAK	Bilayer‐tension gating	Dampens excitability	[107, 108, 109, 110]
Engineered actuators	MscL (WT, I92L, G22S)	High‐tension pore opening	Large‐conductance ion flux	[111, 112]
Tissue‐scale mechanics	Cell–cell junctions, ECM networks	µm‐scale deformation; tissue stress	Coordinates multicellular responses	[39, 130, 131, 132, 133, 134]

## 4 Mechanobiology in Specific Cell Types

Cells across diverse tissues experience unique mechanical schemes that shape their structure, signaling, and function. While the underlying mechanotransduction machinery—integrins, cytoskeletal tension, ion channels, and nuclear deformation—is broadly conserved, its physiological consequences are highly cell‐type‐specific. However, the dominant mechanical inputs and signaling outcomes differ substantially between cell types, shaping how they respond to externally applied mechanical stimulation such as ultrasound.

This section is organized by cell type to support readers who approach sono‐mechanogenetics with a specific biological or therapeutic context in mind. In the context of sono‐mechanogenetics, these differences determine which mechanical interfaces are most effectively engaged by acoustic forces, as well as the relevant deformation modes and stimulation timescales. The following sections briefly highlight key mechanobiological features of representative cell types and discuss their implications for ultrasound‐based actuation.

### 4.1 Neuronal Mechanobiology

Neurons are mechanically soft, excitable cells in which membrane tension and stretch are rapidly converted into electrical, biochemical, and developmental signals. This mechanobiological profile has made the nervous system a primary early testbed for sono‐mechanogenetic control, where mechanically gated ion channels provide a direct route from acoustic perturbation to changes in neuronal activity.

Neurons are intrinsically mechanosensitive and continuously exposed to forces such as membrane tension, shear, and stretch that regulate excitability, morphogenesis, and survival. Piezo channels play central roles in neuronal mechanotransduction. Piezo1 deletion in brainstem or nodose‐ganglion neurons abolishes baroreflexes, demonstrating neuronal stretch sensing for cardiovascular control [138]. Within neural progenitors, substrate stiffness and mechanical stretch evoke Piezo1‐dependent Ca^2^^+^ transients that direct lineage decisions and YAP/TAZ nuclear localization [96, 139]. Piezo1 activation also regulates axon polarization through localized Ca^2^^+^ entry and cytoskeletal remodeling and has been identified as a key mediator of ultrasound neuromodulation, reproducing ultrasound‐evoked neuronal depolarization and behavioral responses in vivo [115, 140]. In contrast, Piezo2 is the principal mechanotransducer for light touch and proprioception, as its conditional deletion in dorsal‐root‐ganglion neurons or Merkel cells abolishes mechanically evoked currents and sensory behavior [99, 141].

Mechanotransduction also defines specialized sensory systems. In cochlear and vestibular hair cells, nanometer‐scale stereocilia deflection activates the TMC1/2 mechanotransduction channel complex, enabling rapid Ca^2^^+^ influx for auditory signaling [142, 143, 144]. Beyond specialized sensory endings, bilayer tension directly gates TRAAK K^+^ channels, stabilizing membrane potential and limiting over‐excitation during osmotic or mechanical stress [107, 145, 146]. At the network level, substrate stiffness programs neuronal morphology and synaptic organization, while cytoskeletal tension and perineuronal‐net mechanics regulate long‐term plasticity and learning [140, 147, 148, 149].

Because neuronal mechanotransduction is dominated by membrane‐localized ion channels with fast gating kinetics, ultrasound stimulation primarily engages membrane tension oscillations that gate mechanosensitive channels, enabling rapid neuromodulation on millisecond timescales. In sono‐mechanogenetics, this positions neurons as optimal targets for temporally precise neuromodulation through direct channel activation and waveform tuning.

### 4.2 Stem Cell Mechanobiology

Stem cells are mechanically plastic cells in which cytoskeletal tension and nuclear mechanics are tightly coupled to long‐term fate decisions and epigenetic state. This coupling makes stem cells particularly attractive for sono‐mechanogenetic strategies aimed at durable “state control,” where transient mechanical inputs can bias differentiation trajectories, regenerative capacity, or functional maturation.

Matrix elasticity strongly influences stem cell lineage specification. Human mesenchymal stem cells cultured on soft, intermediate, or stiff matrices adopt neuronal‐, myogenic‐, or osteogenic‐like fates, respectively, demonstrating that elasticity alone can guide differentiation [150]. Mechanotransduction of these cues proceeds through integrin‐FAK‐actomyosin networks that convert substrate resistance into intracellular tension and nuclear signaling. On stiff matrices, RhoA–ROCK–driven tension promotes nuclear import of YAP/TAZ and MRTF‐A, activating osteogenic transcriptional programs such as RUNX2, whereas cytoplasmic retention on compliant substrates maintains a more undifferentiated, adipogenic, or quiescent state [151].

Beyond elasticity, matrix viscoelasticity and stress relaxation kinetics critically shape stem cell behavior. hMSCs encapsulated in stress‐relaxing alginate or PEG hydrogels exhibit enhanced spreading, actin organization, and bone matrix deposition, outperforming static elastic gels in osteogenic differentiation and mineralization assays [152]. Persistent or cyclic mechanical inputs further induce durable nuclear and epigenetic remodeling, including altered lamin A/C organization, chromatin accessibility, and histone acetylation, which sustain lineage‐biased transcriptional states after force withdrawal [17, 88].

Unlike neurons, stem cells integrate mechanical inputs over longer timescales. Ultrasound in this context is expected to act through cumulative radiation force and low‐level cyclic strain rather than acute membrane gating. Ultrasound stimulation in these cells may preferentially influence cytoskeletal tension and nuclear mechanotransduction, enabling conditioning‐based sono‐mechanogenetic strategies that determine long‐term cell fate decisions rather than immediate responses.

### 4.3 Immune Cell Mechanobiology

Immune cells operate within highly dynamic mechanical environments, migrating across endothelium, squeezing through dense extracellular matrices, and forming transient contacts with target or antigen‐presenting cells. These transitions demand rapid remodeling of cytoskeletal tension and cortical elasticity, allowing immune cells to probe, deform, and respond to physical cues that shape activation, polarization, and effector function.

Innate immune cells show pronounced sensitivity to stiffness and confinement. Macrophages polarize in a stiffness‐dependent manner: rigid substrates enhance spreading, traction, and pro‐inflammatory NF‐κB signaling, whereas compliant environments favor reparative M2‐like states [153]. Geometry alone has also been shown to induce M2 marker expression even without cytokines [154]. Dendritic cells traverse interstitial mazes primarily by actomyosin‐driven “squeezing,” enabling rapid, largely integrin‐independent migration under confinement [155]. Natural killer (NK) cells sense mechanical resistance at the lytic synapse, where increased target or substrate stiffness enhances degranulation and cytotoxicity [156]. Piezo1 functions as a central innate immune mechanosensor, coupling matrix stiffness to Ca^2^^+^ influx, YAP/TAZ and NF‐κB activation, inflammatory polarization, and regulation of cytokine programs that bias T‐cell differentiation within tumors [93, 157].

Adaptive immune cells leverage force for antigen discrimination. T lymphocytes apply piconewton‐scale forces through the TCR to form catch bonds that prolong receptor engagement and amplify signaling [158, 159]. Actomyosin‐driven actin flow further tunes TCR phosphorylation, Ca^2^^+^ signaling, and cytokine output in a stiffness‐dependent manner [40]. Mechanical tension transmitted through LFA‐1 similarly stabilizes ICAM‐1 binding and enhances TCR signaling, while YAP restrains NFAT1 nuclear translocation on soft substrates [160, 161, 162]. B cells likewise use actomyosin pulling to mechanically extract membrane‐bound antigens, favoring high‐affinity interactions during germinal‐center selection [163].

These observations indicate that immune mechanotransduction is strongly context‐dependent and biased toward signaling amplification rather than direct force sensing. In sono‐mechanogenetics, such bias suggests that mechanical stimulation may be most effective when coupled to intracellular signaling circuits that gate activation, differentiation, or immune memory.

### 4.4 Cancer Cell Mechanobiology

Cancer cells exhibit altered and heterogeneous mechanobiology shaped by genetic mutations, cytoskeletal reprogramming, and adaptation to mechanically abnormal microenvironments. Tumor progression is accompanied by ECM stiffening, solid stress accumulation, and elevated interstitial fluid pressure, all of which reshape cancer cell behavior through mechanosensitive signaling networks. Atomic force microscopy reveals that breast, pancreatic, and hepatic tumors are markedly stiffer than normal tissues, reflecting collagen crosslinking and fibrillar alignment that generate anisotropic tension to guide invasion and metastasis [164, 165].

Controlled in vitro systems employing polyacrylamide or polyethylene glycol hydrogels with tunable elasticity have shown that increasing matrix stiffness enhances integrin clustering and focal adhesion assembly, activating FAK–Src and RhoA–ROCK signaling to drive cytoskeletal contractility and growth factor receptor crosstalk [166]. RhoA‐dependent tension recruits YAP/TAZ and, together with nuclear mechanotransduction via lamin A/C and nuclear pore stretching, promotes YAP/TAZ nuclear localization, reinforcing oncogenic mechanical feedback loops [48, 167, 168].

Mechanical signaling in cancer also interfaces with chromatin organization and epigenetic memory. Force‐induced changes in chromatin accessibility and nucleoplasmic protein mobility can persist after mechanical stimulation, providing a form of mechanomemory [88]. In parallel, cellular softness has emerged as a key determinant of tumorigenicity and immune evasion: softer cancer cells exhibit enhanced stemness and self‐renewal mediated by reduced cytoskeletal tension [169], while increased deformability in tumor‐repopulating cells limits perforin‐mediated killing by cytotoxic T cells [170]. Geometric confinement further contributes to genomic instability and invasion through nuclear rupture and coordinated force transmission in aligned ECM architectures [171, 172]. These altered mechanical states suggest that ultrasound perturbations may preferentially engage tumor‐specific mechanotransduction pathways, potentially enabling mechanical targeting of malignant cells or their microenvironment.

### 4.5 Endothelial and Vascular Mechanobiology

Endothelial cells are specialized mechanosensors that integrate shear stress and cyclic stretch to regulate vascular tone, permeability, and inflammatory state. Their continuous exposure to patterned mechanical inputs makes the vasculature a unique context of sono‐mechanogenetic control of transport, barrier function, and inflammatory signaling.

Endothelial cells line the vascular lumen and serve as the primary sensors of mechanical forces generated by blood flow and vessel wall deformation. In vivo, they experience wall shear stress (WSS) ranging from ∼10–70 dyn/cm in arteries and ∼1–6 dyn/cm in veins [173]. Shear sensing is initiated by a mechanosensory complex comprising PECAM‐1, VE‐cadherin, and VEGFR2, which transduces flow into intracellular signaling via Src and PI3K, leading to cytoskeletal remodeling and nitric oxide production [38]. Sustained laminar shear activates Krüppel‐like factors 2 and 4 (KLF2/4), inducing eNOS and thrombomodulin expression while repressing adhesion molecules such as VCAM‐1 and E‐selectin [174, 175]. In contrast, disturbed flow promotes NF‐κB–driven inflammation and actin‐dependent YAP/TAZ nuclear translocation, contributing to endothelial proliferation and barrier dysfunction [176].

Cyclic stretch represents a second key mechanical input. Physiological levels of stretch maintain junctional integrity and vascular homeostasis, whereas excessive strain disrupts endothelial adhesion and increases permeability [177, 178, 179]. Because endothelial cells are adapted to interpret patterned mechanical inputs such as shear and stretch, ultrasound‐induced fluid motion and cyclic strain may provide a means to modulate vascular signaling, permeability, and transport processes.

## 5 Current Applications of Sono‐Mechanogenetics

**FIGURE 3:**
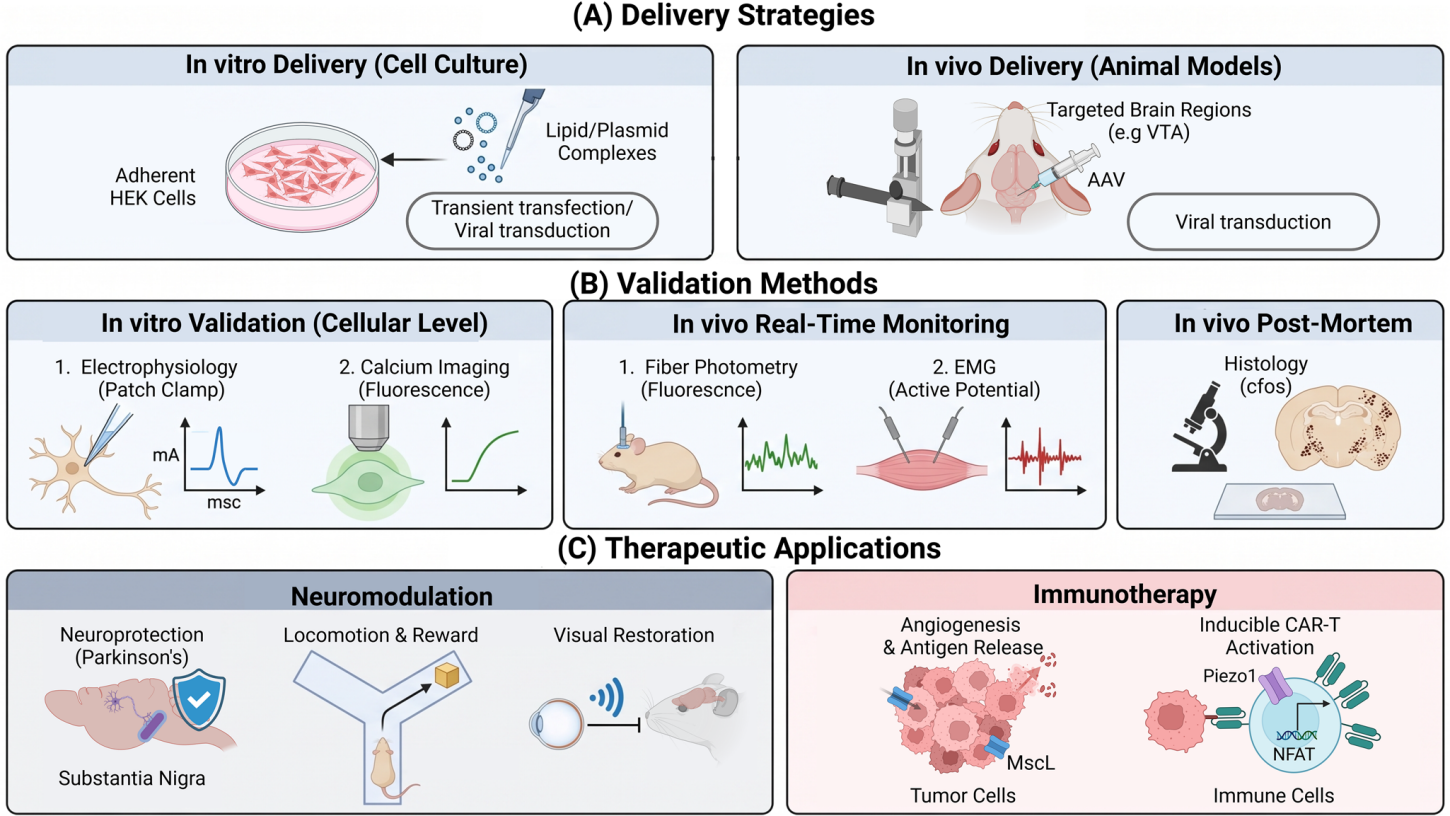
Methodological workflow and applications of sono‐mechanogenetics. (A) Delivery Strategies: Methods for introducing mechanosensitive actuators range from lipid‐mediated transfection for in vitro screening to viral transduction (AAV) or nanogel injection for in vivo targeting. (B) Validation Methods: Ultrasound sensitivity is confirmed via cellular biophysics (patch‐clamp, calcium imaging), real‐time physiological monitoring (fiber photometry, EMG), and post‐mortem activity mapping (c‐Fos). (C) Therapeutic Applications: Validated systems enable precise neuromodulation (neuroprotection, behavioral control, sensory restoration) and cancer immunotherapy (inducing calcium‐mediated apoptosis or activating synthetic CAR‐T gene circuits). **Disclosure Statement**: Initial conceptual layout and draft visual design were generated with assistance from Google Gemini (accessed January 2026). Selected visual components/icons were generated using BioRender AI (accessed January 2026). The figure was subsequently redrawn, refined, arranged, and finalized by the authors using BioRender. All scientific content and visual elements were reviewed and verified by the authors for accuracy and appropriateness.

This section reviews current demonstrations of sono‐mechanogenetics, focusing on systems in which focused ultrasound engages genetically specified mechanosensitive pathways to control cellular function. Rather than organizing studies by therapeutic goal, we group existing work according to the dominant biological interface through which ultrasound‐generated mechanical energy is coupled to cells, including membrane‐associated actuators and endogenous mechanotransduction pathways. This perspective provides a snapshot of the present landscape of sono‐mechanogenetic applications, spanning mature neural modulation paradigms and emerging immunotherapy strategies, while also revealing common mechanistic constraints that shape performance and translational potential (Figure 3). The specific ultrasound parameters and stimulation paradigms utilized across these landmark studies are summarized in Table 3, providing a comparative overview of the acoustic regimes currently employed for in vivo regulation.

**TABLE 3:** Representative ultrasound parameters and stimulation paradigms reported in key sono‐mechanogenetic application studies. The table summarizes major in vitro and in vivo studies in neural modulation and immunotherapy, including actuator/platform, biological model, acoustic frequency, pressure, and stimulation paradigm. Together, these studies illustrate the broad parameter space currently used across sono‐mechanogenetic applications, spanning distinct mechanogenetic actuators, target tissues, and therapeutic goals. Abbreviations: PRF, pulse repetition frequency; PW, pulse width; DFO, duty factor; VTA, ventral tegmental area; STN, subthalamic nucleus; PD, Parkinson's disease; CAR‐T, chimeric antigen receptor T cell.

Actuator/platform	Application	Model	Freq.	Pressure	Stimulation paradigm
mPrestin (N7T, N308S) [181]	Neural modulation	HEK293T cells; primary neurons; mouse VTA	0.5 MHz	0.5 MPa	In vitro: 2000 cycles, 10 Hz PRF, 3 s; In vitro: 1 kHz PRF, 150 cycles, 6 s.
hsTRPA1 [119]	Neural modulation	HEK cells, primary neurons, mouse motor cortex	7 MHz	2.5 MPa for in vitro assays; lower neuronal patch responses around 0.25–0.5 MPa; intact‐skull paradigms up to about 0.88 MPa	In vitro: 100 ms; In vivo: 1/10/100 ms
MscL‐I92L [5]	Neural modulation	Primary rat hippocampal neurons	29.92 MHz	0.12–0.45 MPa; spiking at 0.25 MPa	SAW‐based ultrasound, 1s
MscL‐G22S [185]	Neural modulation	Mouse striatum, VTA, STN; PD models	0.5 MHz, 0.9 MHz	0.05–0.2 MPa in dSTR photometry; 0.1–0.35 MPa in wearable behavior; 0.35 MPa in STN‐PD treatment	Photometry: 400–500 µs PW, 300 ms duration, 1 kHz PRF; Behavior: 00 µs PW, 300 ms, 1 kHz PRF; Treatment: 500 kHz, 400 µs PW, 300 ms, 1 ms pulse interval, 30 min/day for 5 days.
MscL/ MscL‐G22S [123]	Neural modulation/visual restoration	Retinal explants, visual cortex, blind‐animal visual restoration models	0.5, 2.25, 15 MHz	0.11–0.88 MPa at 0.5 MHz; 0.30–1.60 MPa at 2.25 MHz; 0.20–1.27 MPa at 15 MHz	1 kHz PRF, 50% duty cycle, 10–200 ms sonication, 0.01–2 s interstimulus interval
mPrestin (N7T, N308S) [186]	Neural modulation/PD therapy	SH‐SY5Y cells; mouse substantia nigra PD model	0.5 MHz	0.5 MPa	3 s on/7 s off, total 600 s, weekly, for 8 weeks
Piezo1 + MB [98]	Immunotherapy	HEK293T, Jurkat T cells, primary T cells	2 MHz	∼0.6 MPa at the bubble site	5 Hz PRF, 10% duty factor, 5 s for Ca^2^^+^ imaging; 10 min for gene induction
CaDox circuit [189]	Immunotherapy/tumor priming for CAR‐T	PC‐3 organoids and xenografts in a CAR‐T framework	2 MHz in organoids; 1 MHz in vivo	0.63 MPa in organoids; 1.23 MPa in vivo	100 ms pulse duration, 10% DC; organoids: 1 min on/4 min off × 6; in vivo: repeated stimulation over 30 min with 1 min intervals every 2 min

### 5.1 Neural Modulation

Neurons represent the most extensively explored application of sono‐mechanogenetics to date. This prominence reflects not only the biomedical importance of neuromodulation, but also the intrinsic mechanobiology of excitable membranes. Neuronal function is tightly coupled to membrane tension, ion flux, and cytoskeletal dynamics, allowing mechanical perturbations to be rapidly converted into electrical and biochemical signals. As a result, many ultrasound‐based neuronal implementations prioritize fast and reversible responses over durable transcriptional reprogramming, making neural systems a natural proving ground for mechanogenetic actuation.

Early mammalian sonogenetic efforts therefore focused on introducing or sensitizing membrane‐associated actuators to lower the threshold for stimulation. These approaches leverage ultrasound‐induced membrane deformation, radiation force, or tissue displacement to gate ion flux directly, producing millisecond‐ to second‐scale excitation observable at electrophysiological, calcium‐signaling, and behavioral levels. In this sense, neuromodulation has served as the primary proof‐of‐concept domain for demonstrating that focused ultrasound can achieve genetically targeted, on‐demand cellular control.

Neuronal sonogenetics has converged on a small set of representative actuator classes that illustrate distinct strategies for coupling ultrasound to membrane excitability. TRP‐family channels such as C. elegans TRP‐4 can enable behavioral responses to low‐pressure ultrasound when expressed, with additional sensitivity reported in some studies under microbubble coupling [3]. The bacterial mechanosensitive channel MscL has also become a widely used actuator in mammalian systems [111, 112]. To support ultrasound‐evoked spiking, MscL is often sensitized through mutations such as I92L or G22S that lower the mechanical gating threshold [5, 180]. In parallel, mammalian hsTRPA1 has been reported as a broadly ultrasound‐responsive actuator [119]. Its function appears to rely on lipid–cytoskeleton coupling, including cholesterol interactions and actin integrity, suggesting that membrane microdomains and cytoskeletal preload shape gating under ultrasound.

Beyond channels, non‐channel membrane proteins can also confer ultrasound responsiveness. Engineered prestin variants such as mPrestin (N7T/N308S) can produce ultrasound‐evoked calcium signals, potentially via membrane electromechanical coupling rather than canonical pore gating [181]. Alongside exogenous actuators, endogenous mechanosensitive channels, most notably Piezo1, are increasingly implicated in ultrasound‐induced neuronal responses [115, 182, 183]. Although endogenous channels are less orthogonal than engineered actuators, they provide an informative reference point for understanding how native mechanotransduction contributes to ultrasound sensitivity.

Across these implementations, neuronal mechanogenetic actuation is often interpreted through radiation force and tissue displacement as dominant or contributing mechanical routes that couple acoustic energy to membrane tension and ion flux. This linkage, from acoustic exposure to membrane mechanics to excitability, forms the mechanistic backbone for the in vitro and in vivo work summarized below.

#### 5.1.1 In Vitro Methodologies and Validation

Because neuronal sonogenetics was the earliest and most intensively studied application of sono‐mechanogenetics, in vitro systems became the primary arena where actuator mechanisms, measurement modalities, and confounds were debated and refined. Variability across early reports helped motivate a set of experimental practices that now serve as methodological reference points for mechanogenetic studies in other cell types.

A common development pathway follows a practical hierarchy: heterologous expression for rapid screening, followed by validation in primary neurons. Candidate actuators are frequently overexpressed in HEK293 cells due to robust growth and transfection efficiency, enabling fast evaluation of whether ultrasound can be coupled to calcium influx or membrane excitation. This strategy has been central to mechanistic studies of hsTRPA1, where heterologous expression enabled systematic testing of dependencies such as cholesterol content and cytoskeletal integrity [119]. Similar HEK‐based validation has been used for mPrestin constructs, supporting the possibility that membrane electromechanics, not only pore gating, can generate ultrasound‐evoked calcium signals [181].

Promising candidates are then evaluated in primary neuronal cultures to confirm function in a native excitable membrane environment. A representative example is the study by Ye et al., which transduced HEK cells and rat primary neurons with sensitized MscL (I92L) and used patch‐clamp electrophysiology to demonstrate ultrasound‐evoked depolarizing currents and membrane responses compatible with spiking [5]. Subsequent studies extended primary‐neuron validation to additional MscL variants, including MscL‐G22S, and often adopted calcium imaging readouts such as GCaMP to quantify responses across larger populations [152]. These experiments collectively shaped a key lesson: actuator choice and cellular context influence not only sensitivity, but also what is most readily measurable, fast currents versus slower calcium transients.

Readout choice is therefore not purely practical; it shapes mechanistic interpretation. Patch clamp provides the most direct measure of ultrasound‐gated currents and fast channel opening, but it also exposes a critical technical challenge [124]. Acoustic perturbations can destabilize gigaseals and introduce leak currents that resemble mechanogating [15]. This concern, raised explicitly in the literature, contributed to the broader adoption of imaging‐based methods. Calcium imaging using dyes or genetically encoded reporters has become a dominant modality for screening and mapping parameter dependence, with the tradeoff that calcium signals may reflect indirect amplification rather than direct excitability [184].

Beyond cortical neurons, ex vivo or specialized neuronal preparations have been used to test generalizability. For example, MscL variants have been evaluated in retinal neurons and visual pathway models, supporting extension to other neural tissues [123]. Prestin‐based systems have also been explored in neuronal contexts as calcium‐signaling actuators, complementing channel‐centric strategies and broadening the range of membrane‐level coupling mechanisms considered plausible [181].

Across these in vitro studies, the field converged on a shared set of controls aimed at distinguishing mechano‐gating with threshold from secondary effects. Common practices include hydrophone‐based pressure calibration to define the local acoustic field, continuous temperature tracking to exclude inadvertent heating, sham stimulation, and parameter sweeps over pulse width and pressure to identify regimes where cavitation or streaming could confound interpretation. These controls reflect a mechanistic reality: ultrasound can engage multiple physical pathways in parallel, so mechanogenetic claims depend on showing which pathway is dominant under the tested conditions.

#### 5.1.2 In Vivo Applications: From Anesthesia to Awake Behavior

Translation from in vitro systems to intact organisms represented a key inflection point, requiring genetically specified mechanosensitivity to be coupled with ultrasound delivery through complex and heterogeneous tissue. In vivo studies therefore, served not only to demonstrate functional relevance, but also to reveal biological and physical constraints that shape mechanogenetic actuation under realistic conditions.

A typical workflow uses stereotaxic delivery of viral vectors encoding the actuator into a defined neuronal population, followed by a recovery period to allow expression. Ultrasound is then delivered either through a cranial window or transcranially through the intact skull. This distinction matters mechanistically. Craniotomy‐based coupling reduces acoustic aberration and improves the predictability of local pressure delivery, whereas transcranial stimulation must contend with skull‐induced attenuation, scattering, and mode conversion. Even in mice, skull effects can produce substantial discrepancies between nominal transducer output and the local mechanical stimulus experienced by neurons.

Early demonstrations often relied on anesthetized preparations to reduce motion and behavioral variability. One influential study expressed a sensitized prestin variant (mPrestin N7T/N308S) in the ventral tegmental area and applied focused ultrasound under anesthesia, then used post‐hoc c‐Fos mapping as an indirect readout consistent with actuator‐dependent activation [181]. Related work with hsTRPA1 similarly used molecular and histological readouts to argue that ultrasound‐driven membrane mechanics can be converted into spatially restricted excitation in actuator‐expressing regions [119].

As the field matured, in vivo validation expanded toward physiological and behavioral readouts. Fiber photometry became a common tool, allowing real‐time measurement of calcium dynamics in neurons co‐expressing actuators and calcium indicators. This approach strengthened causal links by aligning stimulation epochs with immediate activity changes in the same animals. In parallel, behavioral paradigms began to show that actuator‐dependent stimulation could drive circuit‐level outputs. For example, MscL variants targeted to striatal regions have been associated with ultrasound‐evoked calcium transients and motor behaviors, and targeting dopaminergic populations has been linked to reward‐associated behavioral effects in actuator‐expressing animals [185].

Therapeutic relevance has also been explored in disease contexts where focal circuit dysfunction is well defined, including Parkinson's disease models. Several studies have reported, in preclinical Parkinson's models, that repeated ultrasound stimulation of actuator‐expressing dopaminergic circuits can improve motor phenotypes and may influence degeneration trajectories over multi‐week regimens, raising the possibility that mechanogenetic excitation can engage activity‐dependent survival pathways [186]. Sensory restoration provides another direction. In models of vision loss, expression of MscL variants in retinal neurons or visual circuits has been used to test whether ultrasound can substitute for sensory input in learned behavioral tasks, suggesting that mechanogenetic activation can support functional encoding rather than merely induce nonspecific arousal [123].

Across in vivo studies, several constraints recur. Acoustic field uncertainty increases in intact tissue, particularly for transcranial delivery, making calibration and reproducibility central issues. Expression level and cellular context also shape outcomes, where overexpression can introduce basal currents or cellular burden, and low expression can produce false negatives. Finally, behavioral readouts integrate many downstream processes, raising the need to separate direct mechanogating from indirect network, vascular, or auditory effects. These constraints have motivated more multimodal validation strategies that combine calcium readouts, immediate early gene mapping, electrophysiology, and behavior within the same experimental framework.

Together, in vivo neuronal studies support the core claim of the field: genetically specified mechanical sensitivity can be translated into circuit‐level modulation. At the same time, they emphasize that neuromodulation outcomes reflect the intersection of actuator biology, acoustic delivery, and validation modality, not ultrasound exposure alone.

### 5.2 Immunotherapy

In contrast to neuronal modulation, where the primary objective is rapid and reversible electrical excitation, immunotherapy‐oriented sono‐mechanogenetics is fundamentally concerned with programming cellular behavior over longer timescales. Contemporary cancer immunotherapies, including chimeric antigen receptor (CAR) T cells, immune checkpoint blockade, and synthetic receptor circuits, rely on precise regulation of immune activation, antigen recognition, and spatial specificity. While these approaches have achieved transformative success in hematological malignancies, their extension to solid tumors remains limited by off‐target toxicity, poor tumor selectivity, and lack of spatiotemporal control over immune activation.

Sono‐mechanogenetics offers a distinct opportunity in this context by providing a noninvasive, deeply penetrating, and spatially addressable physical input that can gate immune functions only where and when desired. Unlike neuromodulation, where ultrasound‐induced mechanical perturbations are used to evoke immediate electrophysiological responses, immunotherapy applications typically exploit mechanical stimulation to initiate calcium‐dependent signaling cascades that drive tumor cell death or activate engineered transcriptional programs. In these systems, ultrasound serves as a remote mechanical switch that interfaces with genetically specified mechanotransduction pathways to control immune behavior with spatial precision.

Importantly, the immunotherapy studies described below do not rely on a single form of mechanical input. Instead, distinct implementations engage different modes of ultrasound‐induced mechanical perturbation, including microbubble‐amplified membrane deformation, acoustic radiation force–driven tissue displacement, and membrane tension arising from heterogeneous mechanical microenvironments. Preserving clarity about the operative mechanical mode is essential for interpreting biological outcomes and for translating these strategies across systems.

One direct immunotherapeutic strategy applies mechanogenetic actuation to tumor cells themselves by introducing exogenous mechanosensitive “kill switches.” In this paradigm, ultrasound‐induced mechanical perturbation produces a catastrophic ionic imbalance rather than finely tuned signaling. He et al. delivered E. coli MscL plasmids into tumor cells using an iron–alginate nanogel carrier combined with polyethylenimine, enabling robust expression of the large‐conductance mechanosensitive channel on the tumor cell membrane [187]. Upon ultrasound stimulation, MscL opening drove sustained calcium overload and apoptotic cell death in vivo. The resulting cellular debris promoted tumor‐associated antigen release, dendritic cell maturation, and downstream T‐cell activation, framing this approach as a mechanically triggered form of immunogenic cell death.

A complementary study by Wen et al. employed organelle‐targeted, modified MscL in A549 non–small‐cell lung cancer cells [188]. In this system, ultrasound stimulation biased cells toward vacuolization‐associated, non‐apoptotic death pathways and achieved tumor suppression under low‐intensity focused ultrasound. Across both studies, the unusually large pore size and low gating threshold of MscL allow modest mechanical inputs to be amplified into irreversible ionic and organelle imbalance. Mechanistically, these implementations likely rely on ultrasound‐induced membrane tension and deformation dominated by acoustic radiation force, with potential local amplification arising from nanoparticle carriers or heterogeneous mechanical environments. The precise contribution of cavitation remains incompletely resolved, underscoring the need for more explicit mechanical characterization in future studies.

A second strategy shifts the mechanogenetic target from tumor cells to immune cells themselves, aiming to remotely gate immune activation and effector function. Pan et al. established a general framework for ultrasound‐controlled immune cell programming by engineering HEK293T cells, Jurkat T cells, and primary T lymphocytes to express the mechanosensor Piezo1 together with calcium‐responsive genetic transducer modules [98]. In this system, ultrasound stimulation induced calcium influx, activated calcineurin, and promoted NFAT nuclear translocation, resulting in inducible transcription of user‐defined genes, including CAR constructs.

Crucially, this implementation relied on microbubble‐mediated mechanical amplification. Microbubbles were localized to the cell membrane, where ultrasound‐driven oscillation converted acoustic pressure into large, localized membrane strain and curvature. In this configuration, ultrasound does not directly gate Piezo1. Instead, microbubbles function as mechanical intermediates that transduce acoustic energy into the high‐tension membrane states required for efficient Piezo1 activation. This design explicitly leverages known Piezo1 mechanobiology and provides a clear mapping between acoustic input, mechanical perturbation, and biological sensor. This work demonstrated that immune cell activation could be gated remotely and noninvasively, establishing a blueprint for ultrasound‐controlled adoptive cell therapy. At the same time, it highlighted key constraints of immune‐cell mechanogenetics, including transcriptional latency on the order of hours and the added complexity introduced by microbubble cofactors.

Building on immune‐cell mechanogenetic frameworks while avoiding exogenous mechanical amplifiers, Yoon et al. developed a cofactor‐free platform that exploits intrinsic tumor mechanosensitivity rather than engineered mechanosensitive channels [189]. Focused ultrasound was shown to evoke calcium signaling selectively in invasive cancer cells through endogenous mechanotransduction pathways [190, 191]. This calcium response was then coupled to a doxycycline‐gated AND‐logic synthetic circuit, termed CaDox, enabling spatially confined expression of a clinically validated antigen (CD19) within a subpopulation of tumor cells.

Mechanistically, this approach relies exclusively on acoustic radiation force and bulk tissue deformation to generate membrane tension and cytoskeletal stress, without microbubbles or other local mechanical amplifiers. The mechanical input is therefore weaker but more distributed, engaging native mechanotransduction machinery rather than a single engineered sensor. The induced antigen‐expressing tumor cells functioned as local “training centers” that primed synNotch CAR T recognition and killing in xenografted NSG mouse models bearing engineered PC‐3 tumors. This distinction highlights an important design trade‐off in immunotherapy‐oriented sono‐mechanogenetics. Microbubble‐amplified strategies offer efficient, channel‐specific mechanogating at low acoustic pressures, whereas cofactor‐free approaches rely on distributed native mechanotransduction and favor genetic reprogramming over immediate cytotoxicity. Both paradigms underscore how ultrasound‐induced mechanical inputs can be matched to specific biological architectures to achieve spatially precise immune control.

Collectively, these studies illustrate a spectrum of immunotherapy‐oriented sono‐mechanogenetic strategies, ranging from mechanically triggered tumor cell death to programmable immune‐cell activation and tumor‐local genetic conditioning. Across these implementations, ultrasound serves not as a direct therapeutic agent but as a precision mechanical control input that gates potent biological programs only within defined spatial and temporal windows. Preserving explicit linkage between ultrasound physics, mechanical perturbation mode, and mechanotransduction pathway will be essential for advancing these approaches beyond proof‐of‐concept and toward robust, translatable cancer immunotherapies.

### 5.3 Practical Constraints and Translational Barriers

Despite compelling demonstrations, sono‐mechanogenetics has not yet reached broad translational deployment. This gap reflects practical constraints that complicate reliable implementation in complex in vivo settings, rather than a lack of mechanistic plausibility.

#### 5.3.1 Biological Constraints: Delivery, Scaling, and Cellular Burden

A central biological challenge is efficient and safe delivery of genetic components at scales relevant for translation [192, 193]. In small animal models, localized injection can transduce a substantial fraction of a target population [194]. As tissue volumes increase, whether in larger brains, solid tumors, or distributed immune compartments, achieving adequate coverage becomes increasingly difficult due to diffusion limits and heterogeneity [195, 196].

Cellular burden and long‐term safety are also limiting. Many actuators are derived from non‐mammalian proteins, including bacterial channels such as MscL. While such channels offer favorable gating properties, prolonged expression in mammalian cells can introduce basal leak currents or cytotoxicity, particularly when expression must be sustained for weeks to months. Similar issues can arise from chronic overexpression of highly mechanosensitive mammalian channels, where unintended activation by endogenous forces may perturb normal function [197].

These concerns extend beyond neural applications. In immune cells, basal calcium influx can impair persistence or bias differentiation [198]. In tumors, heterogeneous expression can create uneven response landscapes. Together, these issues motivate strategies that reduce dependence on high‐level chronic expression of a single actuator, favoring approaches that leverage endogenous pathways, transient conditioning, or distributed circuits.

#### 5.3.2 Engineering Constraints: Acoustic Propagation and Mechanical Control

A major engineering barrier is the difficulty of controlling ultrasound‐induced mechanical perturbations in heterogeneous tissue. Propagation depends strongly on tissue composition and boundary conditions, and interfaces such as bone or air cavities distort acoustic fields through reflection, refraction, and phase aberration, producing spatially heterogeneous pressure and force distributions.

This challenge is most prominent in transcranial delivery, where skull thickness and geometry distort beams and attenuate pressures, complicating delivery of well‐defined mechanical stimuli to small targets without unintended heating or standing waves [198]. Similar issues arise in fibrotic tumors and anisotropic tissues, where stiffness heterogeneity shapes how radiation force and displacement distribute at the cellular scale.

Imaging‐based feedback methods such as MR‐ARFI and ultrasound‐based ARFI offer promising routes to monitor mechanical effects in situ, but they are not yet standard in sonogenetic workflows [199, 200, 201]. As a result, many studies still rely on nominal acoustic parameters as proxies for mechanical dose, limiting reproducibility and cross‐study comparison.

#### 5.3.3 System‐Level Constraints: Variability and Context Dependence

A further barrier is that mechanotransduction is strongly context‐dependent. Cellular response depends on matrix properties, confinement, cytoskeletal state, and mechanical history. Identical ultrasound exposures can therefore produce divergent outcomes across tissues, disease states, or even neighboring regions within the same tissue [202, 203].

This variability complicates translation by undermining assumptions of uniform response. In some contexts, mechanical inputs may primarily engage membrane tension and channel gating, while in others they may propagate through cytoskeletal or nuclear pathways [14]. Without explicit consideration of mechanical context and mechanical dose, scaling from simplified models to physiologically complex systems will remain difficult.

Together, these constraints clarify why progress toward predictive control has been slower than early demonstrations might suggest. They also motivate the conceptual framework in Chapter 6, where mechanobiology is treated as a guiding design framework rather than a background context.

## 6 Conceptual Framework and Future Directions

Despite rapid progress in ultrasound‐mediated biological modulation, sono‐mechanogenetics remains at an early and largely exploratory stage. Numerous studies have demonstrated that ultrasound can drive cellular responses when coupled to genetically specified mechanosensitive elements, yet the field has not converged on unifying principles that connect acoustic stimulation to established mechanisms of mechanotransduction. In contrast, decades of mechanobiology research have defined how cells sense, integrate, and adapt to mechanical cues across molecular, cellular, and tissue scales. Bridging these bodies of knowledge represents a critical opportunity to move sono‐mechanogenetics from empirical demonstration toward mechanistically guided and predictive control.

### 6.1 Beyond Single Sensors: Distributed Mechanotransduction

Most current sonogenetic strategies center on identifying or engineering individual mechanosensitive components, most commonly ion channels, that act as primary acoustic sensors. This approach offers molecular specificity and experimental tractability, but it risks oversimplifying how mechanical information is processed in living cells. Mechanobiology has consistently shown that mechanical signals are rarely confined to a single molecular entity. Forces applied at the membrane propagate through integrin‐based adhesions, cytoskeletal networks, and the nucleus, engaging multiple layers of mechanosensitive machinery in parallel. Ion channels such as Piezo or TRP family members often serve as early responders, yet their gating depends strongly on membrane tension, cytoskeletal prestress, and cellular architecture rather than isolated force sensing. In parallel, mechanical deformation of the nucleus can directly influence chromatin organization and transcription, in some cases bypassing canonical biochemical signaling pathways. These observations suggest that ultrasound‐delivered mechanical energy cannot be assumed to act locally or exclusively on engineered actuators.

Rather than viewing this distributed coupling as a limitation, future sonogenetic designs can exploit it. Systems that intentionally engage cytoskeletal tension, nuclear deformation, or chromatin accessibility may achieve more robust and scalable responses than those relying on a single molecular trigger. In this view, engineered sensors function less as isolated switches and more as entry points into a broader mechanotransduction network.

### 6.2 Time as a Design Variable in Sono‐Mechanogenetics

A defining insight from mechanobiology is that cells encode not only the magnitude of applied forces, but also their temporal structure. Transient forces can elicit rapid signaling events such as calcium influx or adhesion turnover, whereas sustained or repetitive loading drives cytoskeletal reinforcement, transcriptional reprogramming, and long‐term phenotypic change. Importantly, forces of identical amplitude can produce qualitatively different outcomes depending on duration, frequency, and prior mechanical history. Ultrasound offers exceptional control over these temporal dimensions, including pulse duration, repetition rate, and duty cycle. Yet many sonogenetic studies still frame responses in binary terms, activated versus not activated, without systematically exploring how temporal patterns shape downstream biology. Treating ultrasound as a time‐structured mechanical signal, rather than a simple on–off trigger, opens the possibility of encoding graded, sequential, or memory‐dependent cellular programs.

Mechanomemory further extends this concept. Mechanical cues can induce persistent cellular states through cytoskeletal remodeling, nuclear adaptation, and epigenetic modification. While such persistence is often viewed as a confounding factor, it may instead be leveraged as a design feature. Brief, instructive ultrasound exposures could prime cells into durable functional states, reducing the need for continuous stimulation and minimizing off‐target effects.

### 6.3 Quantifying Mechanical Dose and Context

A major obstacle to progress in sono‐mechanogenetics is the lack of standardized metrics linking ultrasound parameters to biologically relevant mechanical forces. Acoustic intensity, frequency, and pressure are often reported without reference to the strain, stress, or deformation experienced by cells. In mechanobiology, however, force magnitude and deformation regime are central to interpreting biological outcomes. Without quantitative mapping between acoustic parameters and mechanical inputs, it remains difficult to compare results across studies or to relate ultrasound‐driven responses to known mechanotransduction pathways. This challenge is compounded by the strong context dependence of mechanosensitivity. Cellular responses to force are shaped by extracellular matrix stiffness, confinement, cytoskeletal organization, and nuclear mechanics, meaning that identical ultrasound exposures can elicit divergent outcomes across tissues or disease states.

Future work must therefore prioritize the development of frameworks that translate ultrasound exposure into approximate mechanical descriptors compatible with mechanobiological understanding. Even coarse estimates of deformation mode or force regime would significantly improve reproducibility and cross‐study comparison. Incorporating mechanical context into experimental design will be essential for translating sono‐mechanogenetics into complex in vivo environments.

### 6.4 Toward Mechanically Guided Ultrasound Control

The central challenge for sono‐mechanogenetics is not technological feasibility, but conceptual alignment. The field will advance most rapidly by treating mechanobiology not as background knowledge, but as a guiding framework for tool development. Ultrasound physics defines the space of mechanical inputs that can be generated, while mechanobiology determines how those inputs are sensed, integrated, and remembered by cells.

By embracing distributed mechanotransduction, temporal encoding, and mechanical context, future sonogenetic systems can move beyond empirical activation toward rational, predictive control of cellular behavior. Such an approach has the potential to transform sono‐mechanogenetics from an emerging technique into a foundational modality for remote, noninvasive regulation of biology.

## 7 Conclusion

Sono‐mechanogenetics has emerged at the intersection of ultrasound physics, mechanobiology, and genetic engineering as a promising strategy for remote, noninvasive control of cellular function. Unlike optical or chemical modalities, ultrasound delivers mechanical energy deep within tissues and across spatial scales that are directly relevant to endogenous mechanotransduction pathways. Early demonstrations have established feasibility across neural modulation, immunotherapy, and synthetic gene regulation, yet the field remains heterogeneous in both mechanism and implementation.

In this review, we have framed sono‐mechanogenetics through the lens of mechanobiology, emphasizing that ultrasound‐driven responses cannot be understood solely in terms of acoustic parameters or isolated molecular actuators. By integrating physical principles of ultrasound propagation with cell‐type–specific mechanotransduction and experimental applications, we highlight that mechanical context, temporal structure, and distributed force transmission fundamentally shape biological outcomes. This perspective moves beyond cataloging ultrasound‐responsive tools and instead situates sono‐mechanogenetics within a broader framework of how cells naturally sense, integrate, and remember mechanical information.

Looking forward, progress in sono‐mechanogenetics will depend less on discovering ever more sensitive actuators and more on achieving conceptual alignment across disciplines. Mechanistically informed design, quantitative mapping between acoustic exposure and cellular deformation, and careful consideration of mechanical context will be essential for reproducibility and translation. As ultrasound technologies continue to mature and genetic tools become increasingly programmable, the opportunity lies in developing systems that leverage, rather than bypass, endogenous mechanotransduction networks. If pursued in this manner, sono‐mechanogenetics has the potential to evolve from an exploratory technique into a foundational modality for precise, scalable, and noninvasive control of biological systems.

## Conflicts of Interest

Y. Wang is a scientific co‐founder and consultant of Cell E&G Inc. and Acoustic Cell Therapy Inc. These financial interests do not affect the design, conduct, or reporting of this research. The other authors declare that have no conflicts of interest.

## Data Availability

Data sharing not applicable to this article as no datasets were generated or analysed during the current study.
